# Navigating the Neurobiology of Migraine: From Pathways to Potential Therapies

**DOI:** 10.3390/cells13131098

**Published:** 2024-06-25

**Authors:** Masaru Tanaka, Bernadett Tuka, László Vécsei

**Affiliations:** 1 HUN-REN-SZTE Neuroscience Research Group, Danube Neuroscience Research Laboratory, Hungarian Research Network, University of Szeged (HUN-REN-SZTE), Tisza Lajos krt. 113, H-6725 Szeged, Hungary; tanaka.massaru.1@med.u-szeged.hu; 2 Department of Radiology, Albert Szent-Györgyi Medical School, University of Szeged, Semmelweis u. 6, H-6725 Szeged, Hungary; tuka.bernadett@med.u-szeged.hu; 3 Department of Neurology, Albert Szent-Györgyi Medical School, University of Szeged, Semmelweis u. 6, H-6725 Szeged, Hungary

## 1. Introduction

Migraine is a debilitating neurological disorder characterized by recurring episodes of throbbing headaches that are frequently accompanied by sensory disturbances, nausea, and sensitivity to light and sound [1,2,3,4]. With a global prevalence of approximately 15% and a significant impact on individuals’ quality of life, migraine represents a major public health concern [5,6,7]. Despite its high prevalence and impact, the underlying mechanisms of migraine remain complex and multifaceted, involving a combination of genetic, environmental, and neurobiological factors [8,9,10]. Understanding the pathophysiology of migraine is essential for developing targeted therapies that can effectively manage symptoms and improve patient outcomes [11,12,13].

Recent advances in migraine research have emphasized the importance of experimental models in understanding the neurobiological mechanisms that cause migraine attacks [14,15,16,17,18]. Experimental models, particularly murine models, have provided valuable insights into the molecular and cellular pathways involved in migraine pathophysiology, allowing researchers to investigate the neurotransmission, inflammation, and sensitization processes that underpin migraine attacks [19,20,21,22]. Researchers have identified key molecules, pathways, and neural circuits that contribute to the initiation and progression of migraine attacks by simulating migraine conditions in controlled experimental settings [23,24,25]. These experimental models not only enhance our understanding of migraine pathophysiology but also serve as valuable tools for testing potential therapeutic targets and interventions.

The pathophysiology of migraines is significantly influenced by neuroplasticity, which includes modifications to brain excitability, biochemistry, and functional connectivity [26,27,28]. Chronic migraines are linked to persistent changes in neural plasticity, such as central sensitization and impaired pain modulation mechanisms [29,30,31,32,33]. These changes can set off a vicious cycle of pain chronification, in which the brain’s reaction to pain becomes maladaptive and pathological. Several receptors, including calcitonin gene-related peptide (CGRP), transient receptor potential vanilloid subtype 1 (TRPV1), and purinergic receptor P2X subtype 3 (P2X3), have also been implicated in migraine mechanisms. For example, CGRP has been shown to stimulate trigeminal afferent activity and sensitize nociceptive neurons, whereas TRPV1 and P2X3 receptors are involved in mechanical pain modulation and nociceptive signal generation, respectively. Understanding the interplay between neuroplasticity and receptor activity is essential for developing targeted therapies for neurological disorders, including migraine [34,35,36,37].

The following Special Issue, entitled “Migraine Neuroscience: From Experimental Models to Target Therapy”, aims to showcase the latest research findings and advancements in the field of migraine neuroscience, with a specific focus on experimental models and therapeutic targets for migraine treatment. This collection of articles brings together cutting-edge studies that explore the intricate interplay of the neurobiological processes involved in migraine, from neurotransmitter signaling to neuroinflammation and genetic predisposition. By bridging the gap between basic science research and clinical applications, this Special Issue seeks to provide a comprehensive overview of the current state of migraine research and offer insights into novel therapeutic strategies for managing this complex neurological disorder.

## 2. Topic Articles

### 2.1. Calcitonin Gene-Related Peptide (CGRP)-Related Mechanisms and Therapies

CGRP plays a pivotal role in migraine pathophysiology [38,39,40]. During both migraine attacks (ictal phase) and headache-free periods between attacks (interictal phase), CGRP levels are elevated [41,42,43]. However, after abortive and prophylactic treatment, these levels tend to decrease [44,45]. Notably, CGRP can induce migraine-like headaches in patients, and studies have demonstrated its significant release during acute migraine and cluster headache attacks [46,47,48]. Chronic migraine may be associated with chronically elevated CGRP levels [49,50,51]. Beyond headache mechanisms, CGRP also contributes to maintaining a normal resting tone in brain circulation [52,53,54]. Perivascular CGRP appears to mediate a protective vasodilatory reflex triggered in response to vasoconstriction [55,56,57]. Additionally, trigeminal pathway activation leads to the release of both CGRP and substance P [58,59,60]. A literature review highlights the variability in study design, determination methods, and results for CGRP measurements in migraine patients [61]. Overall, the above findings collectively emphasize the importance of CGRP in migraine and highlight the need for standardized measurement methods, potentially informing therapeutic strategies targeting CGRP for migraine treatment.

#### 2.1.1. Interaction between Calcitonin Gene-Related Peptide (CGRP) and Nitric Oxide in Migraine

Benedicter et al. investigated the complex relationship between glycerol trinitrate (GTN) and CGRP in the context of trigeminal nociception, a key mechanism in migraine pathogenesis [62]. The researchers used rodent experiments and the anti-CGRP antibody Fremanezumab to better understand GTN’s downstream effects on CGRP signaling. Their findings provide compelling evidence that GTN acts downstream of CGRP in the trigeminal nociceptive system, suggesting that the modulation of CGRP is a critical factor in the complex pathways that drive migraine pain. By unraveling this interplay between two key players in migraine, the authors contribute valuable insights that may inform the development of targeted therapeutic strategies for migraine management. The findings of their study underscore the importance of understanding the intricate mechanisms underlying migraine in order to develop more effective and personalized treatments for this debilitating condition.

Greco et al. investigated the interaction between CGRP and pain mediators involved in neuronal sensitization in an animal model of chronic migraine [63]. The study authors aimed to investigate the central and peripheral mechanisms of CGRP receptor antagonism. The authors revealed that olcegepant reduced the incidence of trigeminal hyperalgesia by lowering the expression of CGRP in the trigeminal nucleus and proinflammatory markers such as cytokines, microRNA-132, and TRPA1. The results suggest that CGRP receptor antagonism is important in both the central and peripheral mechanisms of chronic migraine. The results of the above study have the potential to contribute to the development of novel therapeutic strategies for chronic migraine, potentially leading to improved treatment outcomes and enhanced patient quality of life.

Dux et al. investigated the effects of Fremanezumab, an anti-CGRP monoclonal antibody, on CGRP release from the rat dura mater and meningeal blood flow [64]. In the rat model, the authors found that administering Fremanezumab reduced CGRP release from the dura mater and, as a result, meningeal blood flow. These findings shed light on the mechanisms by which anti-CGRP antibodies, such as Fremanezumab, can modulate CGRP-mediated processes critical to migraine pathogenesis. The above study advances our understanding of the therapeutic potential of CGRP-targeted interventions in migraine management by elucidating Fremanezumab’s effect on CGRP release and meningeal blood flow (Table 1).

#### 2.1.2. Real-World Outcomes and New Therapeutic Targets for Migraine

Pavelic et al. conducted a systematic review to assess the real-world outcomes of monoclonal antibodies targeting CGRP for migraine prophylaxis [65]. CGRP has been recognized as a key player in migraine pathophysiology, and the development of CGRP-targeted therapies, including monoclonal antibodies, has revolutionized migraine management. In their review, the authors analyzed data from 134 publications, including retrospective and clinic-based studies, case reports, and other articles. The study findings suggest that treatment with anti-CGRP monoclonal antibodies is associated with lower healthcare utilization, better treatment adherence, and comparable efficacy to randomized controlled trials. The authors do, however, acknowledge that the retrospective study designs, small patient populations, and short follow-up periods used in the examined studies limit the availability of real-world data. They emphasize the need for large prospective studies with long-term follow-up to fully understand the real-world impact of these novel therapies. By synthesizing the available evidence on the efficacy and safety of anti-CGRP monoclonal antibodies in clinical practice, the authors contribute to the growing body of knowledge on the practical implications of these therapies in migraine management.

Tanaka et al. addressed the potential of other neuropeptides, such as pituitary adenylate cyclase-activating polypeptide (PACAP) and vasoactive intestinal peptide (VIP), to better understand the evolving landscape of migraine treatment beyond CGRP [66]. These neuropeptides have shown promise in novel migraine management as a potential therapeutic target in addition to CGRP. Their review delves into the transition from CGRP to PACAP, VIP, and beyond, shedding light on the next generation of migraine treatments. By unraveling the roles of these neuropeptides in migraine pathophysiology, the authors contribute to expanding the understanding of potential targets for migraine therapy beyond the traditional focus on CGRP. Their research paves the way for exploring novel treatment avenues and advancing the field of migraine management (Table 1).

### 2.2. Metabolic Pathways and Migraine

Altered tryptophan (Trp) metabolism plays a crucial role in migraine susceptibility through its main metabolites, serotonin and kynurenine (KYN), which affect pain processing, stress response, neural hypersensitivity, and inflammatory processes [74,75,76,77,78,79,80,81,82]. These pathways have been extensively studied in the context of migraine, with a focus on their influence on vascular and inflammatory mechanisms [68,83,84,85]. The involvement of Trp metabolism, particularly through the serotonin and KYN pathways, has been a key area of research in understanding migraine pathophysiology [86,87]. The authors of recent studies have identified potential therapeutic targets within these pathways for future drug development, emphasizing the importance of regulating Trp-KYN metabolism in migraine treatment [88,89]. The intricate interplay between neurotransmitters, neuropeptides, and inflammatory mediators underscores the complexity of migraine pathogenesis and the potential for targeted interventions to improve treatment outcomes [90,91].

#### 2.2.1. Altered Tryptophan Metabolism and Migraine Susceptibility

Gecse et al. investigated the neuroendocrine response to citalopram in patients with migraine [67]. The authors used a neuroendocrine challenge to assess the metabolism of Trp and KYN, a key amino acid and its metabolite involved in migraine pathophysiology. The findings of their study indicate that patients with migraine exhibit altered Trp-KYN metabolism compared to healthy controls. Specifically, they observed increased Trp levels and decreased KYN levels in migraine patients. These changes suggest that the metabolism of these amino acids may play a crucial role in the development and continuation of migraine. By elucidating the neuroendocrine response to citalopram in migraine patients, the results of the above study advance our understanding of the complex mechanisms that underpin migraine, highlighting the potential therapeutic implications of targeting Trp-KYN metabolism in migraine management (Table 1).

#### 2.2.2. Tryptophan Metabolism Pathways in Migraine: Therapeutic Implications

Körtési et al. present a narrative review that explores the role of Trp metabolism in migraine pathogenesis [68]. The authors looked at the complex interactions of Trp metabolic pathways and how they affect migraine-related mechanisms such as pain processing, stress response, neural and brain hypersensitivity, and vascular and inflammatory processes. Their review highlights the importance of Trp metabolism in migraine susceptibility and the potential therapeutic implications of targeting these pathways. By synthesizing the current knowledge of Trp metabolism in migraine, they provide a comprehensive overview of the underlying mechanisms and identify promising avenues for future drug development and migraine management strategies (Table 1).

### 2.3. Experimental Models and Therapeutic Targets

Experimental models and therapeutic targets are critical for better understanding migraine pathophysiology and developing effective treatments [92,93,94]. Experimental models, particularly murine models, provide invaluable insights into the biological mechanisms that underpin migraines, allowing researchers to test various pharmacological interventions [16,95]. These models simulate migraine conditions, allowing researchers to study neurotransmission pathways and identify key molecules involved in migraine attacks. Therapeutic targets identified by the authors of such studies provide promising treatment options, focusing on molecules and pathways that can be modulated to alleviate migraine symptoms [12,96,97,98]. The authors of recent studies have identified several potential targets, including enzymes, ion channels, and signaling pathways, each of which contribute uniquely to our understanding of migraine and offer new opportunities for therapeutic intervention [88,99,100,101,102]. Three papers delve into the specifics of these experimental models and therapeutic targets, providing a thorough overview of current progress and future directions in migraine research [69,70,71].

#### 2.3.1. Dual Fatty Acid Amide Hydrolase (FAAH)/Monoacylglycerol Lipase (MAGL) Inhibitor in the Migraine Model

Greco et al. investigated the potential therapeutic benefits of targeting the endocannabinoid system in the context of migraine pathophysiology [69]. Utilizing a male rat model, the authors examined the effects of trigeminal hyperalgesia using AKU-005, a dual fatty acid amide hydrolase (FAAH) and monoacylglycerol lipase (MAGL) inhibitor. The study reported that administering AKU-005 significantly reduced trigeminal hyperalgesia in the rat model, implying that dual inhibition of FAAH and MAGL could be a useful strategy for managing migraine-related pain. By elucidating the effects of AKU-005 on trigeminal hyperalgesia, the findings of the above study contribute to the growing body of research on the role of the endocannabinoid system in migraine and highlight the potential therapeutic implications of this novel pharmacological intervention for migraine management.

#### 2.3.2. Src Family Kinase (SFK) Activity and Calcitonin Gene-Related Peptide (CGRP)–Cytokine Crosstalk

Nie et al. investigated the role of Src family kinases (SFKs) in the interaction between CGRP and cytokines in sensitizing the trigeminal ganglion [70]. CGRP and cytokines are key players in migraine pathophysiology, and their interaction is thought to contribute to the sensitization of the trigeminal system, a crucial mechanism underlying migraine-related pain. The study authors revealed that SFKs play a crucial role in mediating the crosstalk between CGRP and cytokines in the trigeminal ganglion. Specifically, they demonstrated that SFKs facilitate the transmission of CGRP receptor signaling via the protein kinase A pathway, leading to the sensitization of trigeminal ganglion neurons. By elucidating the mechanisms by which SFKs regulate the interaction between CGRP and cytokines in the trigeminal system, the authors contribute to our understanding of the complex pathways involved in migraine pain and highlight the potential therapeutic implications of targeting SFKs in migraine management.

#### 2.3.3. ATP-Sensitive Potassium (KATP) Channels in Migraine Pathophysiology

Clement et al. explored the potential role of ATP-sensitive potassium (KATP) channels in migraine pathophysiology and their therapeutic implications [71]. KATP channels are known to be involved in various physiological processes, including vascular regulation and pain perception, making them an intriguing target for migraine research. The researchers synthesized findings from both preclinical and clinical studies to provide a comprehensive overview of the current understanding of KATP channels in migraine. The above review emphasizes the translational potential of KATP channel modulation in migraine management, with the results of animal studies indicating that KATP channel openers may have anti-nociceptive effects and reduce the incidence of migraine-related behaviors. Additionally, genetic studies involving humans have identified associations between KATP channel subunits and migraine susceptibility. By integrating these findings, Clement et al. propose that targeting KATP channels could be a promising avenue for the development of novel migraine therapies. Their review underscores the importance of translational research in bridging the gap between preclinical discoveries and clinical applications in the field of migraine treatment (Table 1).

### 2.4. Inflammation in the Pathophysiology of Migraine

Inflammation plays a pivotal role in the pathophysiology of migraine [1,103,104,105,106]. The complex mechanisms underlying migraine include neurogenic inflammation, which contributes to peripheral and central sensitization, resulting in the condition’s chronicity [107,108,109,110]. Two papers included in the present Special Issue examine the current understanding of neurogenic neuroinflammation and its role in migraine pathophysiology, focusing on how inflammatory processes in the meninges and trigeminal nerve pathways cause and sustain migraine pain [72,73]. Additionally, the authors of the second paper explore the wider symptomatology linked to migraines, highlighting the significance of recognizing and managing symptoms other than headache, such as photophobia and nausea, which have a major influence on patients’ quality of life [73]. The following section provides a brief overview of the inflammatory processes involved in migraine, as well as potential strategies for mitigating these effects in order to improve patient outcomes.

#### 2.4.1. Neurogenic Neuroinflammation in Migraine

Reducha et al. explored the potential of using experimental inflammation models to gain a better understanding of migraine pathophysiology [72]. Their review highlights how, in addition to other mechanisms, neurogenic neuroinflammation has been proposed to play a role in the chronification of migraine, which involves peripheral and central sensitization. Extensive data indicate that CGRP and intracranial meningeal inflammation may play a major role in triggering the sensitivity of trigeminal meningeal nociceptors. The authors discuss how several studies have utilized inflammatory animal models to investigate this concept, with a focus on the sensitization of trigeminovascular afferent nerve terminals. By applying a range of pharmacological interventions, these studies provide insights into the pathways involved in the inflammatory processes underlying migraine. However, the authors emphasize the importance of using animal models with care and carefully evaluating the outcomes in the context of migraine pathophysiology, as the invasive procedures used in these models may have implications for data interpretation. Overall, the above review underscores the potential of experimental inflammation models in enhancing our understanding of the complex mechanisms driving migraine while also highlighting the need for cautious interpretation and translation of the findings into a clinical setting.

#### 2.4.2. Complex Symptomatology of Migraine

Villar-Martinez and Goadsby conducted a comprehensive review of the pathophysiology and treatment of migraine-associated symptoms [73]. Their review highlights that migraine is a complex and heterogeneous disorder that goes beyond the core symptom of headache. The authors discuss the various associated features, including photophobia, vomiting, and other symptoms that are often overlooked but can significantly impact patients’ quality of life. The review authors emphasize the importance of understanding these associated features in the context of migraine pathophysiology as they can provide valuable insights into the underlying mechanisms driving the disorder. Additionally, the authors discuss the therapeutic implications of these associated features, including the potential for targeted interventions to alleviate symptoms and improve patient outcomes. By synthesizing the current understanding of migraine-associated features, the authors of the above review provide a comprehensive overview of the complex interplay between migraine pathophysiology and therapy, highlighting the need for a more holistic approach to managing this debilitating disorder.

## 3. Discussion

Migraine research has undergone significant advancements in recent years, shedding light on the intricate neurobiological mechanisms that underlie this debilitating neurological disorder [111,112,113]. The present Special Issue on migraine neuroscience serves as a comprehensive compilation of cutting-edge research in the field, with a specific emphasis on experimental models and therapeutic targets aimed at enhancing our understanding of migraine pathophysiology and improving patient outcomes. The ultimate goal of this line of research is to develop targeted therapies that can effectively manage migraine symptoms and alleviate the burden of this complex disorder on individuals’ quality of life. By delving into the molecular and cellular pathways involved in migraine attacks, researchers strive to identify novel therapeutic strategies that can address the diverse array of symptoms experienced by migraine sufferers, ranging from throbbing headaches to sensory disturbances, nausea, and sensitivity to light and sound [66].

However, a significant challenge in the field of migraine research lies in bridging the gap between basic science discoveries and their translation into clinical applications. To overcome this challenge, researchers must collaborate across disciplines, integrating knowledge from neuroscience, genetics, pharmacology, and other fields to develop innovative treatment modalities. Leveraging cutting-edge technologies such as advanced imaging techniques, genetic sequencing, and computational modeling is crucial in unraveling the complexities of migraine pathophysiology and identifying potential therapeutic targets. For example, the functional interplay between error-related brain activity and the autonomic nervous system in migraine emphasizes the significance of coordinated interactions between the central and autonomic nervous systems [114,115]. These interactions are critical for survival because they aid in the detection and correction of errors that could be fatal [116,117,118].

Advancements in migraine research have not only deepened our understanding of the underlying mechanisms driving migraine attacks but have also paved the way for personalized and targeted treatment approaches. These studies expand on previous research on CGRP-related mechanisms, Trp metabolic pathways, and experimental models, providing new insights and having implications for future research directions in migraine neuroscience [119,120,121]. Traditional medicine, particularly those derived from herbal compounds, has proven to be effective in the treatment of a variety of neurological and mental health disorders [122,123,124,125,126,127]. These remedies have played an important role in the field, and ongoing research is being conducted to identify potential antimigraine agents [128,129,130]. Herbs and other traditional forms of treatment have been linked to the treatment of conditions such as migraine [131,132,133,134,135,136]. By investigating the properties of these herbs, researchers hope to develop more effective medicines and targeted treatments for various conditions, including migraine. Furthermore, neurophysiological procedures have emerged as promising non-invasive interventions for migraine treatment, such as transcranial magnetic stimulation (TMS), with the potential to reduce migraine frequency and severity by modulating cortical excitability and inhibiting cortical spreading depression. [137,138,139,140]. TMS has a lower side effect profile than traditional pharmacological treatments, making it an appealing option for patients who cannot tolerate medication [141,142].

The significance of this line of research extends beyond the realm of academia, with profound implications for clinical practice and patient care. By elucidating the neurobiological underpinnings of migraine and exploring novel therapeutic avenues, researchers aim to revolutionize the management of this complex neurological disorder, providing tailored treatment options that address the individual needs and symptoms of migraine sufferers. Furthermore, the collaborative efforts of basic science researchers and clinicians are essential in driving forward the field of migraine research and translating scientific discoveries into tangible benefits for patients. By embracing multidisciplinary approaches and innovative research methodologies, the study of migraine neuroscience holds enormous promise for future breakthroughs and improved outcomes for individuals affected by this debilitating condition.

## 4. Conclusions

The following Special Issue on migraine neuroscience has shed light on the intricate mechanisms underlying this debilitating neurological disorder. Through experimental models and therapeutic targets, researchers have made significant strides in understanding migraine pathophysiology, from genetic predisposition to neurotransmitter signaling and neuroinflammation. The emphasis on CGRP-related mechanisms and the exploration of Trp metabolic pathways have provided valuable insights into potential treatment avenues. Moving forward, future research directions could focus on further unraveling the complex interplay of neurobiological processes involved in migraine, including investigating novel therapeutic strategies and exploring the impact of environmental factors on migraine development. Collaborative efforts between basic science researchers and clinicians will be crucial in filling the gap between research findings and clinical applications, ultimately leading to improved management of this complex neurological disorder. By continuing to push the boundaries of our understanding and embracing multidisciplinary approaches, the field of migraine research holds enormous promise for innovative breakthroughs and improved outcomes for migraine sufferers.

## Figures and Tables

**Table 1:** Major subtopics covering the Special Issue “Migraine Neuroscience: From Experimental Models to Target Therapy”.

Subtopics		Ref.
1. CGRP-related mechanisms and therapies		
	a. Interaction between CGRP and nitric oxide in migraine	[62,63,64]
	b. Real-world outcomes and new therapeutic targets for migraine	[65,66]
2. Metabolic pathways and migraine		
	a. Altered tryptophan metabolism and migraine susceptibility	[67]
	b. Tryptophan metabolism pathways in migraine: therapeutic implications	[68]
3. Experimental Models and Therapeutic Targets		
	a. Dual FAAH/MAGL inhibitor in a migraine model	[69]
	b. SFK activity and CGRP–cytokine crosstalk	[70]
	c. KATP channels in migraine pathophysiology	[71]
4. Inflammation and pathophysiology in migraine		
	a. Neurogenic neuroinflammation in migraine	[72]
	b. Complex symptomatology of migraine	[73]

CGRP: calcitonin gene-related peptide; FAAH: fatty acid amide hydrolase; KATP: ATP-sensitive potassium; MAGL: monoacylglycerol lipase; SFKs: Src family kinases.

## Abbreviations

CGRP	Calcitonin gene-related peptide
FAAH	Fatty acid amide hydrolase
GTN	Glycerol trinitrate
KATP	ATP-sensitive potassium
KYN	Kynurenine
MAGL	Monoacylglycerol lipase
PACAP	Adenylate cyclase-activating polypeptide
P2X3	Purinergic receptor P2X 3
SFKs	Src family kinases
TMS	Transcranial magnetic stimulation
Trp	Tryptophan
TRPV1	Transient receptor potential vanilloid subtype 1
VIP	Vasoactive intestinal peptide
